# Effects of long-term dehydration on stress markers, blood parameters, and tissue morphology in the dromedary camel (*Camelus dromedarius*)

**DOI:** 10.3389/fvets.2023.1236425

**Published:** 2023-12-05

**Authors:** Mahmoud A. Ali, Hassan Abu Damir, Muna A. Adem, Osman M. Ali, Naheed Amir, Asma A. M. Shah, Salama S. M. Al Muhairi, Khaled O. S. Al Abdouli, Javed R. Khawaja, Tareq A. Fagieri, Abdelnasir Adam, Aboubakr A. Elkhouly, Zhaya J. Al Marri, Mohamed Jamali, David Murphy, Abdu Adem

**Affiliations:** ^1^Department of Pharmacology, College of Medicine and Health Sciences, United Arab Emirates University, Al-Ain, United Arab Emirates; ^2^Veterinary Laboratory Division, Animal Wealth Sector, Abu Dhabi Food Control Authority, Abu Dhabi, United Arab Emirates; ^3^Department of Biochemistry, Khawarizmi College, Al-Ain, United Arab Emirates; ^4^Molecular Neuroendocrinology Research Group, Bristol Medical School: Translational Health Sciences, University of Bristol, Bristol, United Kingdom; ^5^Department of Pharmacology, College of Medicine and Health Sciences, Khalifa University, Abu Dhabi, United Arab Emirates

**Keywords:** dromedary camel, dehydration/rehydration, oxidative stress, antioxidant vitamins, stress hormones/catecholamine, reproductive hormones, diagnostic enzymes

## Abstract

**Introduction:**

Dromedary camels robustly withstand dehydration, and the rough desert environment but the adaptation mechanisms are not well understood. One of these mechanisms is that the dromedary camel increases its body temperature to reduce the process of evaporative cooling during the hot weather. Stress in general, has deleterious effects in the body. In this study, we sought to determine the effects of dehydration and rehydration on stress parameters in the dromedary camels and how it pacifies these effects.

**Methods:**

Nineteen male camels were randomly divided into control, dehydrated and rehydrated groups, and fed alfalfa hay *ad-libitum*. The dehydrated and rehydrated groups were water-restricted for 20 days after which the rehydrated camels were provided with water for 72 h. The control and dehydrated camels were slaughtered at day 20 from the start of experiment whereas the rehydrated group was killed 72 h later. Many biochemical, hematological histopathological parameters and gene analysis were performed in relevant tissues collected including blood, plasma, and tissues.

**Results and discussion:**

It was observed that severely dehydrated camels lost body weight, passed very hard feces, few drops of concentrated urine, and were slightly stressed as reflected behaviorally by loss of appetite. Physiologically, the stress of dehydration elicited modulation of plasma stress hormones for water preservation and energy supply. Our results showed significant increase in cortisol, norepinephrine and dopamine, and significant decrease in epinephrine and serotonin. The significant increase in malondialdehyde was accompanied with significant increase in antioxidants (glutathione, retinol, thiamin, tocopherol) to provide tissue protection from oxidative stress. The physiological blood changes observed during dehydration serve different purposes and were quickly restored to normality by rehydration. The dehydrated/rehydrated camels showed reduced hump size and serous atrophy of perirenal and epicardial fat. The latter changes were accompanied by significantly increased expression of genes encoding proteins for energy production (ANGPTL4, ACSBG1) from fat and significantly decreased expression of genes (THRSP; FADS 1&2) encoding proteins enhancing energy expenditure. This process is vital for camel survival in the desert. Dehydration induced no major effects in the vital organs. Only minor degenerative changes were observed in hepatic and renal cells, physiological cardiomyocyte hypertrophy in heart and follicular hyperplasia in splenic but lipidosis was not depicted in liver hepatocytes. Ketone bodies were not smelled in urine, sweat and breathing of dehydrated animals supporting the previous finding that the ß hydroxybutyrate dehydrogenase, a key enzyme in ketone body formation, is low in the camel liver and rumen. Rehydration restored most of blood and tissues to normal or near normal. In conclusion, camels are adapted to combat dehydration stress and anorexia by increasing anti-stressors and modulating genes involved in fat metabolism.

## Introduction

Camels are well adapted to the hot weather in desert and semi-desert environments with their dry, scarce woody shrubs—an environment that could be very difficult for other domestic animals to tolerate. One of the mechanisms enabling the dromedary camel to tolerate heat is an increase in body temperature to reduce the evaporative cooling during the hot weather of the day.

The circulatory system of the camel is resilient to conditions of dehydration as well as rapid rehydration compared to other species. When dehydrated camels are rehydrated, they can drink up to 110 liters of water within 10 min to restore one-third of their body weight loss (1, 2). Adaptive mechanisms such as red blood cell (RBC) shape and elasticity and the high serum albumin concentrations during dehydration play major roles in maintaining osmotic pressure and tissue water conservation (3). Such mechanisms are also operative in other desert species, such as the desert rat (4). The RBCs of the dehydrated camels can swell up to 240% of the initial volume without rupture. However, it is not clear if this large increase in RBC volume in response to rehydration affects their passage through the narrow endothelial gaps of the venous sinuses in the spleen red pulp. In other species, RBCs can only swell by 150% (5).

In addition, aquaporin 1, 2, 3, 4, solute carrier proteins and cholesterol in the kidney cortex and medulla of dehydrated camels have been suggested to play essential roles in water economy in concert with vasopressin, angiotensin, and other peptide hormones (6–8).

Unlike other species, when a dehydrated camel drinks large quantities of water, it stores the water for up to 24 h in the gut with slow absorption to avoid rapid dilution of the circulatory system, thus allowing equilibrium to be maintained very slowly (9).

Normally, the camel uses microbial digestion in the stomach more efficiently than other ruminants and increases serous salivary gland secretions, which act as food diluent and buffer to optimize the rumen environment for bacterial and ciliated protozoan fermentation (5). However, it has been reported that dehydrated camels limit their food intake and reduce metabolic rate and salivary flow (10–15). Previous reports indicate that the dehydrated camels became emaciated but could still move around (16). However, we observed that when the camels became severely dehydrated, they became less active, perhaps to avoid water and energy wastage. Dehydrated animals seemed to rely on body fat mobilization in the form of triglycerides to be catabolized into fatty acids to compensate for energy shortage prior to protein catabolism (17). Body fat is prioritized over protein, as one gram of fat provides nine calories, whereas one gram of protein provides only four calories (18).

Similar situations of anorexia or/energy shortage and stress or/dehydration in other species, such as cattle, sheep, and camelid species, have been reported to instigate ketosis and/or liver lipidosis (19–22). However, in the dromedary camel, ketosis has never been known to occur, and liver lipidosis has not been investigated or reported as a macroscopic finding or perceived microscopically.

This study is part of a large project investigating the dromedary camel tolerance to extended dehydration and quick rehydration. In previous reports, we studied the effect of sustained dehydration with and without angiotensin II AT1 receptor blockade on complete blood count (CBC), serum electrolytes, enzymes, and neuro-hormones (23, 24). Besides, we studied the effects of dehydration and/rehydration on the kidney cortex and medulla. Long-term dehydration provoked some pro-inflammatory and anti-inflammatory cytokines, oxidative stress (OS) antioxidant markers, and apoptosis in the kidney cortex, along with gene expression supporting these findings (25). The kidney medulla was less affected than the cortex during the dehydration and rehydration periods. The effect of cholesterol levels was also investigated in dehydrated and dehydrated/rehydrated camels. Down-regulation of membrane cholesterol levels in kidney cells helped in preserving water during dehydration (7). Contrary to Mesoamerican disease in humans, no kidney fibrosis was observed, but only minor electron microscopic changes were identified in different parts of kidney nephrons [unpublished data]. The effect of long-term dehydration on the abomasum (6) was also reported. The changes observed included cellular vacuoles and focal necrosis in the gastric mucosa, activation of oxidative stress markers, and incited apoptosis via an extrinsic pathway. The gastric glands and endocrine cell secretions were regulated toward water and energy conservation in the dehydrated camels (6).

Water is regulated by arginine vasopressin (AVP) and oxytocin (OXT) produced in the supraoptic nucleus of the hypothalamus. Dehydrated camels showed a significant increase in circulating angiotensin II, AVP, OXT, and upregulation of respective genes along with *CCKAR* gene expression. Transcriptomic study revealed extracellular matrix remodeling of the supraoptic nucleus with enhanced protein processing to augment AVP and OXT syntheses as an adaptive change to protect the animal from dehydration (26).

The dromedary camel is well adapted to the stress of dehydration in desert conditions; however, the mechanisms involved are not yet fully understood. In this study, we provide insights on how dromedary camels ease the stress of dehydration. We hypothesize that the dehydrated dromedary camel developed advanced adaptation mechanisms to forbear the stress of long-term dehydration and the accompanied energy shortage with quick physiological and biochemical adjustments, with little or no effects on vital organs. In this study, we aimed to provide comprehensive biochemical, hematological, and histopathological data on dromedary camels in response to long-term dehydration and subsequent rehydration. The data will be very informative to those working with this species, including camel owners, field veterinarians, researchers, and nutritionists. Moreover, the results further extend our understanding of the pathophysiology of long-term dehydration in dromedary camels.

## Materials and methods

### Ethical approval and adherence to ARRIVE guidelines

The study protocol was approved by the Animal Ethics Committee of the United Arab Emirates University with approval ID No: ERA- 2016-4327. During the experimental period, all applicable international, national, and/or institutional guidelines for the care and use of animals were followed. We would like to confirm that this work adhered to the ARRIVE list of guidelines for reporting animal research.

Nineteen healthy male dromedary camels, 4–5 years old, with a body weight range of 340–375 kg, were used in this study. After a short adjustment period, the camels were divided into the following three groups: control group (*n* = 5), dehydrated group (*n* = 8), and rehydrated group (*n* = 6). The control group was supplied with water *ad libitum* for the whole experimental period; the dehydrated group was deprived of water for 20 days, while the rehydrated group had restricted access to water for 20 days and then allowed free access from day 21 for 72 h. The camel groups were housed separately in a large, well-shaded area that allowed free movement during the months of April and May, near the UAE University, Al-Ain City, the United Arab Emirates. The camels were fed alfalfa hay *ad libitum* for the whole period of the experiment, as a usual practice in all camel farms in UAE. Concentrates were not provided to avoid struvite stones, as observed in practice. The area was usually cleaned on a daily basis with the removal of all food that remained from the previous day. The animals were kept under close veterinary supervision during the experimental period. No obvious clinical signs or symptoms of any disease, such as abnormal sounds of pain, grunting, grinding of teeth, and extension of the neck, were noticed; however, it was observed that the dehydrated camels passed very hard feces and a few drops of concentrated urine. Further, slight distress was also observed in the dehydrated and/rehydrated camels, reflected behaviorally by slightly reduced activity and loss of appetite— they did not consume food offered to them as they did during the start of the experiment or as controls—and physiologically by increased cortisol, tumor necrosis factor (TNF), and interleukins (25, 27, 28).

The control and the dehydrated groups were killed on day 20 of dehydration, whereas the rehydrated group of camels were killed 72 h after. Body weights for all groups were calculated at the baseline date and every five days thereafter using the formula live weight (kg) = shoulder height x chest girth x hump girth x 50 (29).

At the start and end of experiments and before taking the animals to the slaughterhouse, their blood, feces, and skin scraping were tested in our laboratory for main camel diseases. The blood was negative for trypanosomiasis, anaplasmosis, babesiosis, brucellosis, anthrax, and hemorrhagic septicemia. The feces were negative for internal parasites, and skin scrapings were negative for mange and external parasites. At the end of the experimental periods, the camels were taken to the abattoir. In the abattoir, all camels were checked by the veterinarians and passed the ante-mortem and post-mortem checks. During ante-mortem, the camels did not show abnormal gait, loud breathing, abnormal posture, cachexia, edema, anasarca, stomatitis, protrusions, skin diseases, contusions, or neurological signs. Besides, there were neither abnormal secretions nor/excretion from body orifices, abnormal body smells, nor color changes in mucosa or/conjunctiva were observed. Thereafter, the animals were quickly sacrificed. During the post-mortem, the head, eyes, masticatory muscles, mandible, tongue, esophagus, diaphragm, rumen, reticulum, abomasum, spleen, small and large intestines, lung surfaces, heart, kidneys, salivary glands, and internal and external surfaces of the carcass were examined and no abnormality or lesions were observed. Further, the lymph nodes were palpated, incised, and examined. These included parotid, medial, and lateral retropharyngeal; mesenteric, cranial, middle, and caudal mediastinal; hepatic, right, and left bronchial; internal iliac, and superficial inguinal lymph nodes. None of these were swollen, hemorrhagic, containing pus, or showing any other abnormality.

The blood and tissues were immediately collected by a big team of technical staff and veterinary pathologists who handled the specimens carefully in the correct preservatives with minimal jeopardy to quality, and these were sent to the laboratory for further processing. The meat of the slaughtered camels was not wasted; it was either used for human consumption (control camels) or animal consumption (dehydrated and rehydrated camels donated to the Al-Ain Safari Zoo).

### Specimen collection and processing

Blood samples were collected by jugular venipuncture from all groups in plain, heparinized, and anticoagulant (K3-EDTA) vacutainers. Blood collection was performed on day 0, 5, 10, 15, and 20 of dehydration and from the rehydrated group on 12, 24, 48, and 72 h after rehydration for different purposes. Kidney samples were collected within 1 h after slaughtering the camels, immersed immediately in liquid nitrogen, and kept at −80°C. Specimens were shipped on dry ice to the University of Bristol under the auspices of a DEFRA Import License (TARP/2016/063) for gene analysis. Representative pieces from the right liver lobe, right kidney, spleen, parotid salivary gland, and heart left ventricle were promptly fixed in 10% (v/v) neutral buffered formalin pending processing.

### Histopathological methods

We performed histopathology to check for temporary (e.g., degeneration) or permanent (e.g., necrosis) effects induced by dehydration or rehydration. The fixed specimens were processed in an automatic tissue processor (ATP1-220, Triangle Biomedical Sciences, INC., Durham, USA) and embedded in paraffin wax blocks, cut into 5 micrometers thick sections in a microtome (RMT 202A, Bioevopeak Co., Ltd Jinan, Shandong, China). The sections were stained with hematoxylin-eosin routine stain (H&E; Thermo Fisher Scientific; UK) according to a previously described technique (30). The slides were visualized under the light microscope (Olympus digital microscope U-APT, Olympus, Tokyo, Japan).

### Hematological methods

Containers with K3-EDTA anticoagulant were slightly and gently mixed and analyzed using a fully automatic hematology analyzer (Sysmex XT 2000 IV system-ABBOTT- USA). Complete blood count (CBC), including red blood count (RBC), hematocrit (HCT), hemoglobin concentration (Hb), mean corpuscular volume (MCV), mean corpuscular hemoglobin (MCH), mean corpuscular hemoglobin concentration (MCHC), and white blood cell count (WBC) were determined.

### Biochemical methods

The blood, collected in plain vacutainers, was allowed to clot, and the serum was separated by centrifugation at 3,000 rpm, and aliquots in clean tubes were kept at −20°C pending analyses. Blood samples collected in heparinized vacutainers were kept in an ice box and transported within 30 min, centrifuged (1,400 rpm) at +4°C, and the plasma stored as aliquots at −80°C until analyzed.

Biochemical analyses including serum total protein (TP), albumin, calcium (Ca), phosphorus (P), iron (Fe), copper (Cu), alanine aminotransferase (ALT), aspartate aminotransferase (AST), lactic dehydrogenase (LDH), and gamma-glutamyl transferase (GGT), were performed using fully automatic chemistry analyzer (Roche – Lab Cobas- C501- USA). Serum retinol (Vit. A), thiamin (Vit. B1), and tocopherol (Vit. E) were analyzed using high-performance liquid chromatography (HPLC) (Waters Alliance – USA). Plasma-free triiodothyronine (fT3), free thyroxine (fT4), testosterone, progesterone, and cortisol were determined using (Roche – Lab Cobas- E601- USA), while insulin-like growth factor 1 (IGF-1) and insulin-like growth factor binding protein 3 (IGFBP-3) were measured by radioimmunoassay (RIA) (Peninsula Laboratories, CA, USA). Catecholamine was extracted from plasma and measured using HPLC, as described previously (31). Glutathione (GSH) content was estimated according to the method described by the suppliers of the assay kit (Sigma –Aldrich 3050 Spruce St, St. Louis, MO 63103).

The liver homogenates and GSH assay were performed as below:

### Liver homogenate preparation

The liver homogenate was prepared as described earlier (6). Briefly, liver tissue was washed with ice-cold phosphate buffer saline (PBS). Tissue was weighed and homogenized with a complete protease inhibitor cocktail to stop tissue degradation (hydrolysis) by protease enzymes. The resulting homogenates were centrifuged, and the supernatant was stored at −80°C until assayed.

### GSH assay

Sigma-Aldrich Glutathione Assay Kit catalog # CS0260 was used to perform the GSH assay. The preparation of samples and assay were performed following the manufacturer's protocol for the assay kit. The kit contained all the reagents required for deproteinization of the samples and calorimetric assay of total glutathione [oxidized (GSSG) + reduced GSH]. The kit was used to measure the level of total glutathione (GSSG + GSH) in liver tissue extracts. The samples were first deproteinized with a 5% 5-sulfosalicylic acid solution. The glutathione content in the samples was then assayed using a kinetic assay in which catalytic amounts of glutathione cause a continuous reduction of 5,5′-dithiobis-(2-nitrobenzoic) acid (DTNB) to 5-thio- 2-nitrobenzoic acid (TNB) and the oxidized glutathione disulfide (GSSG) formed was then recycled to GSH by glutathione reductase (GR) and nicotinamide adenine dinucleotide phosphate hydrogen (NADPH). The rate of TNB formation was proportional to the sum of GSH and GSSG present in the sample. The yellow color product (TNB) was determined spectrophotometrically at 412 nm within 5 min of DTNB addition against a blank with no homogenate.

The level of malondialdehyde (MDA) from the plasma and tissue of each group was measured using the MDA Assay kit (Northwest Life Science Specialties, LLC 16420 SE McGillivray, Suite 103, PMB 106, Vancouver, WA 98683, USA). The assay is based on the reaction of MDA with thiobarbituric acid (TBA) to form an MDA-TBA2 adduct that exhibits strong absorption at 532 nm. In brief, 250 μl of deproteinized tissue sample was added to 250 μl of 1M phosphoric acid and 250 μl of butylated hydroxytoluene in ethanol, and then the mixture was heated at 60°C for 60 min. The suspension was cooled to room temperature and centrifuged at 10,000 rpm for 2–3 min, and the pink-colored supernatant was taken for spectroscopic measurements at 532 nm for the assay of MDA.

### Statistical analysis

Our objective in this study was to show if there was any difference in the different parameters between control and dehydrated dromedary camels at day 20. To compare the difference in body weight between controls and dehydrated dromedary camels, we applied two-way ANOVA where the first factor was group (control, dehydrated) and the second factor was time (day 0, day 20). In this ANOVA, we added the interaction time between group and time. This interaction was expected to show if the difference in body weight between day 20 and day 0 was the same in the control compared to the dehydrated camels. Similarly, if this interaction in body weight in control and dehydrated camels was the same at day 0 compared to day 20.

Another objective of this study was to investigate whether rehydration for 72 h could reverse the different parameters tested back to normal levels after a long period of dehydration. To meet this objective, we compared the different parameters of the rehydrated camels (*n* = 6) at day 23 to the parameters of control (*n* = 5) and dehydrated (*n* = 8) camels at day 20 using Welch's *t*-test for each comparison. The significance of the means between the two groups was determined by the *t*-test. Means of data with the standard error (Mean ± SEM) and median with IQR were presented. *P*-values < 0.05 were considered significant. Data was analyzed using SPSS software (32).

## Results

### Body weight

There was no significant difference in body weight between all groups at the start of the experiment. The mean body weight (Mean ± SEM) of the control group increased significantly at day 20 compared to day 0. However, the mean body weight of the dehydrated group decreased significantly on day 20 of dehydration, constituting 31% weight loss from their initial mean body weight (Table 1). 72 h after rehydration, the mean body weights of the rehydrated camels increased by 17.9% to 294.5 ± 13 kg compared to the mean body weights of the same camels (249.7 ± 5.4 kg) at day 20 of dehydration. However, the increase in body weight after rehydration was significantly lower (p < 0.001) than the weight of the controls.

**Table 1:** Body weight (BW) (kg) changes in control (days 0 and 20) and dehydrated (days 0 and 20) dromedary camels.

**Groups**	**Day 0 (initial BW)**	**Day 20 (final BW)**
Control	356.8 ± 4.59	364.8 ± 3.14^***^
	358 (17)	365 (12.00)
Dehydrated	363.6 ± 3.57	251.8 ± 8.87^***^, ^**†*††***^
	368 (12.00)	256.00 (46.50)

Data are shown as mean ± SEM and median (IQR) for the control and dehydrated camels. There were no significant differences in the weights of the control and dehydrated groups at day 0. The body weights of dehydrated camels at day 20 were significantly different from those of control at day 0 (^***^p < 0.001) and day 20 (^†*††*^p < 0.001). IQR, interquartile range.

### Gross and histopathological findings

**Figure 1:**
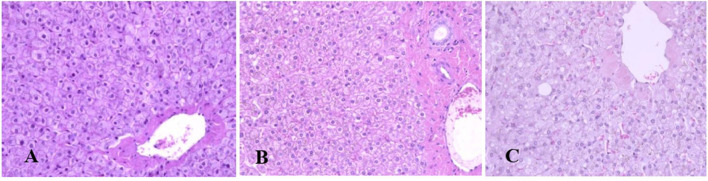
Photomicrographs of the liver tissues of control, dehydrated, and rehydrated camels. No obvious histopathological changes were observed in the liver of controls **(A)**. The dehydrated group **(B)** showed a mild degree of degenerative changes, but no hepatic lipidosis was evident. **(C)** The liver of the rehydrated group revealed mild degenerative changes and focal cytoplasmic vacuoles and necrosis (H&E × 400).

**Figure 2:**
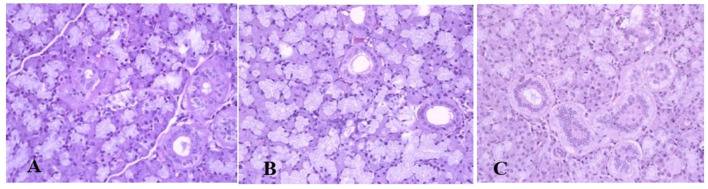
Photomicrographs of the parotid salivary gland of control, dehydration, and rehydration camels. No histopathological changes were observed in the parotid gland tissue of the control group **(A)**. Some acini of parotid glands of dehydrated camels were completely devoid of serous secretions **(B)**. The acini and tubules of the parotid gland of rehydrated camels showed increased serous secretions with pinkish staining of the acini and tubule spaces **(C)**. H & E-staining (magnificatio*n* = 400×).

**Figure 3:**
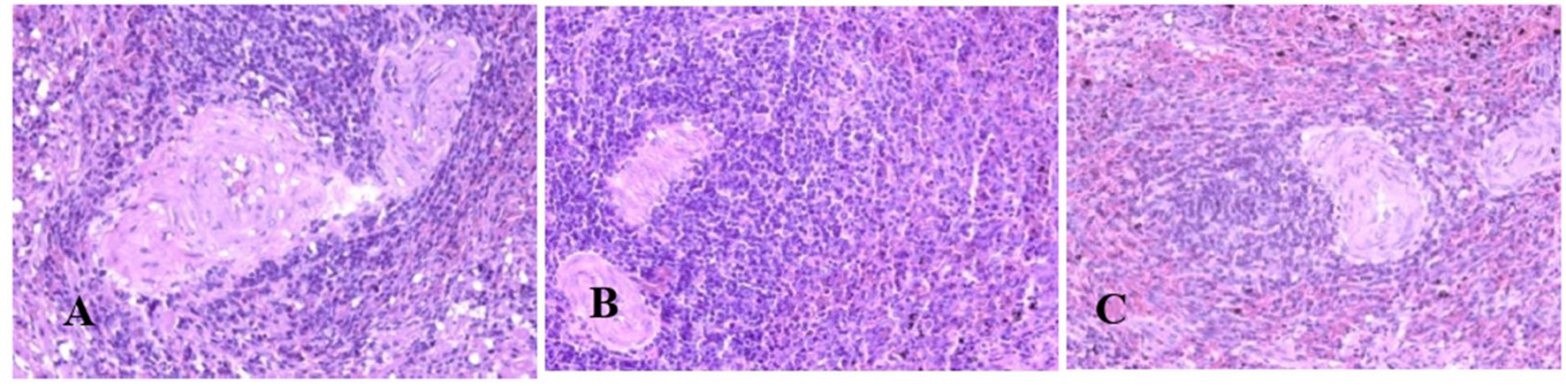
Photomicrographs of the spleen of control, dehydrated, and rehydrated camels. No obvious histopathological changes were observed in the spleen of the control group **(A)**. The dehydrated group depicted a mild lymphocytic hyperplasia in white pulp **(B)**. In the rehydrated group, the red pulp area increased compared with the white pulp of the control and dehydrated groups **(C)**. H & E-staining (magnificatio*n* = 400×).

**Figure 4:**
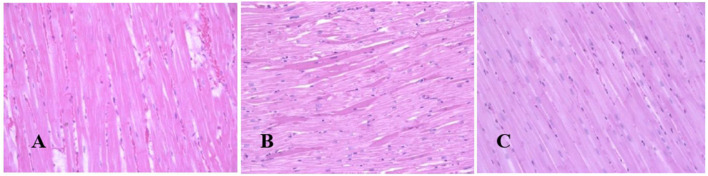
Photomicrographs of the heart tissues of camels after dehydration and rehydration. No obvious histopathological changes were observed in the camel heart tissue of the control group **(A)**. No obvious histopathological changes were observed in the dehydrated group except for a few cardiomyocytes displaying physiological hypertrophy **(B)**. No obvious histopathological changes were noted in the heart tissue of the rehydrated camels except that the tissue retained more dim staining compared to dehydrated and control groups **(C)**. H & E-staining (magnificatio*n* = 400×).

Generally, the camel carcasses showed no abnormalities in all organs during post-mortem except for smaller hump size. Slight serous atrophy of perirenal and epicardial fat was observed in the dehydrated and rehydrated groups. In the dehydrated camels, there was a mild degree of degenerative changes, including hydropic degeneration along with hepatic vacuoles, which was similarly depicted in the liver of rehydrated camels. In addition, focal necrosis, small extracellular nuclear fragments, and pale nuclear and cytoplasmic staining were evident in the hepatocytes of the rehydrated camels compared to the dehydrated and controls. No hepatic lipidosis was observed grossly or microscopically in the livers of all groups (Figure 1). No apparent histopathological changes were observed in the parotid salivary gland (a serous tubuloalveolar) of all camel groups (Figure 2). However, some parotid acini of the dehydrated group were completely devoid of serous secretions, whereas the parotid acini and tubules of the rehydrated group contained serous secretions characterized by pinkish color compared to the blue color seen in the gland's tissue of the controls. In the spleen, mild lymphocytic hyperplasia was seen in the white pulp of the dehydrated camels, increased red pulp area in rehydrated camels, and no obvious histopathological changes were observed in both spleen regions of the controls (Figure 3). Few cardiomyocytes displayed physiological hypertrophy in the dehydrated group. On the other hand, no obvious histopathological lesions were noticed in the cardiac cells of the controls and rehydrated camels. However, the cardiomyocytes of the rehydrated camels retained more dim staining compared with that of the dehydrated and control groups (Figure 4). The kidney histological and electron microscopy changes were mild and mostly degenerative in nature and are presented in another report (Abu Damir et al., unpublished data).

### Oxidative stress (OS) markers

Long-term dehydration induced a significant increase of OS biomarkers in the liver GSH (*p* < 0.05), MDA (*p* < 0.05), and plasma MDA (*p* < 0.01) of the experimental camels. However, short-term rehydration significantly reversed the situation by reducing liver GSH (*p* < 0.01), MDA (*p* < 0.05), and plasma MDA(*p* < 0.05) values to match those of controls (Table 2).

**Table 2:** Liver GSH and MDA and plasma MDA of the control (day 20), dehydrated (day 20), and rehydrated (day 23) camels.

**Parameter**	**Control camels**	**Dehydrated camels**	**Rehydrated camels**
Liver GSH (μm/l)	6,112.36 ± 848.51	8,687.14 ± 666.61^*^	3,941.69 ± 1,010.80^††^
	6,448.17 (3,097)	8,179.36 (2,875)	4,224.71 (3,848)
Liver MDA (μm/l)	2.20 ± 0.41	12.79 ± 3.47^*^	6.07 ± 2.86
	2.10 (1.70)	14.92 (12.00)	5.75 (10.89)
Plasma MDA (μm/l)	2.63 ± 0.18	6.01 ± 0.53^**^	3.67 ± 0.62^†^
	2.48 (0.75)	6.23 (1.98)	3.31 (2.41)

Values are mean ± SEM and median (IQR) for the control, dehydrated, and rehydrated camels. Significant difference from control is denoted by ^*^p < 0.05 and ^**^p < 0.01 and from dehydrated camels by ^†^p < 0.05 and ^††^p < 0.01. GSH, glutathione; MDA, malondialdehyde; IQR, interquartile range.

### Stress hormone markers

Stress hormone data are presented in Table 3. Long-term dehydration caused a significant increase in plasma cortisol (*p* < 0.001), norepinephrine (*p* < 0.05), and dopamine (*p* < 0.05) and a significant decrease in epinephrine (*p* < 0.05), serotonin (*p* < 0.001), IGF-1 (*p* < 0.001), and IGFBP-3 (*p* < 0.01) levels. Rehydration reduced cortisol (*p* < 0.05), epinephrine (*p* < 0.01), norepinephrine (*p* < 0.01), and dopamine (*p* < 0.01) levels, with a significant increase in serotonin (*p* < 0.001) compared with dehydration values. Rehydration further reduced IGF-1 levels compared to both control and dehydrated groups (*p* < 0.05). IGFBP-3 levels were not affected in rehydrated, compared to dehydrated camels.

**Table 3:** Effects of long-term dehydration and short-term rehydration in plasma cortisol, epinephrine, norepinephrine, dopamine, serotonin, IGF1, and IGFBP-3 levels in control (day 20), dehydrated (day 20), and rehydrated dromedary camels (day 23).

**Parameter**	**Control camels**	**Dehydrated camels**	**Rehydrated camels**
Cortisol (nmol/l)	3.84 ± 0.141	26.40 ± 1.99^***^	3.20 ± 0.33^†*††*^
	3.59 (0.83)	24.01 (6.90)	2.76 (1.38)
Norepinephrine (ng/ml)	0.240 ± 0.0181	0.179 ± 0.004^*^	0.071 ± 0.007^***††^
	0.23 (0.04)	0.18 (0.02)	
Epinephrine (ng/ml)	0.130 ± 0.005	0.126 ± 0.006	0.064 ± 0.030^***††^
	0.131 (0.03)	0.12 (0.03)	
Dopamine (ng/ml)	0.218 ± 0.0078	0.360 ± 0.030^**^	0.154 ± 0.024^††^
	0.214 (0.05)	0.34 (0.59)	
Serotonin (ng/ml)	0.320 ± 0.102	0.020 ± 0.001^***^	0.221 ± 0.078^†*††*^
IGF1(ng/ml)	385.13 ± 20.5	107.4 ± 13.7^***^	35.75 ± 6.0^***†*††*^
	382.50 (111.75)	109.00 (76.25)	40.00 (20.25)
IGFBP-3 (ng/ml)	401.50.7 ± 11.54	316.10 ± 6.46^***^	302.00 ± 6.99^***^
	391.00 (73.50)	315.50 (40.50)	297.50 (25.00)

Values are mean ± SE and median (IQR) for the control, dehydrated, and rehydrated camels. Significant difference from control is denoted by ^*^p < 0.05, ^**^p < 0.01, and ^***^p < 0.001, and from dehydrated camels (day 20) is denoted by ^††^p < 0.01 and ^†*††*^p < 0.001. IGF1, Insulin-like growth factor 1; IGFBP-3, Insulin-like growth factor binding protein 3; IQR, interquartile range.

### Hematological findings

Hematological data are presented in Table 4. The results revealed significant increases (*p* < 0.001) in HCT following dehydration, which was sustained for up to 12 h after rehydration compared to the control. Thereafter, rehydration caused a significant decrease (*p* < 0.001) in HCT to match control values. Hb was significantly increased (*p* < 0.01) by dehydration compared to the control. However, Hb levels decreased by rehydration to match those of control. Dehydration caused a non-significant increase in RBC count compared to the control, and gradually, the RBC values returned to control levels after 12 h of rehydration. Dehydration caused a significant increase (*p* < 0.001) in MCV compared to control, and values remained high at 12, 24, 48, and 72 h after rehydration (*p* < 0.001, *p* < 0.01, *p* < 0.05, and *p* < 0.05, respectively). MCH showed a significant increase with dehydration and at 12, 24, and 72 h post rehydration (*p* < 0.01) compared to the control. On the other hand, MCHC showed a significant decrease caused by dehydration (*p* < 0.05) and 12 h (*p* < 0.01) post rehydration. Thereafter, MCHC values increased and matched those of the control group. WBC showed a significant increase (*p* < 0.001) following dehydration, but the values gradually reduced with rehydration to attain control levels by 48 and 72 h.

**Table 4:** Changes in HCT, Hb, RBC, MCV, MCH, MCHC, and WBCs values in control (day 20), dehydrated (day 20), and rehydrated (day 20 and after 12, 24, 48, 72 h after rehydration) camels.

**Parameter**	**Control**	**Dehydrated**	**Rehydrated 12 h**	**Rehydrated 24 h**	**Rehydrated 48 h**	**Rehydrated 72 h**
HCT %	28.1 ± 0.6	33.9 ± 0.5^***^	32.8 ± 0.7^**^	29.8 ± 0.7^†*††*^	30.0 ± 0.7^†*††*^	30.9 ± 0.6^**††^
	28.30 (3.75)	33.95 (2.10)	32.20 (4.05)	29.55 (2.68)	29.95 (3.53)	30.30 (2.27)
Hb g/dl	13.3 ± 0.3	14.9 ± 0.4^**^	14.4 ± 0.4	13.47 ± 0.4^†^	13.6 ± 0.4	14.3 ± 0.4
	13.30 (2.60)	15.30 (2.00)	14.2 (1.85)	13.40 (1.55)	13.60 (1.17)	14.15 (1.97)
RBCs (10^6^/μl)	9.1 ± 0.2	9.8 ± 0.3	9.2 ± 0.4	8.6 ± 0.3	9.1 ± 0.3	9.2 ± 0.2
	9.18 (2.04)	10.18 (1.67)	9.30 (1.24)	8.71 (1.21)	9.25 (0.63)	9.19 (1.04)
MCV (fl)	31.2 ± 0.5	34.2 ± 0.7^***^	35.6 ± 0.7^***^	34.5 ± 0.7^**^	33.4 ± 0.7	33.7 ± 0.6^*^
	30.70 (3.40)	34.10 (3.63)	35.45 (2.30)	34.50 (2.23)	33.15 (2.15)	33.70 (2.23)
MCH (pg)	14.6 ± 0.1	15.4 ± 0.2^**^	15.6 ± 0.3^**^	15.6 ± 0.3^**^	15.2 ± 0.2	15.6 ± 0.3^**^
	14.6 (0.70)	15.40 (1.10)	15.50 (0.85)	15.40 (0.75)	15.10 (0.85)	15.35 (1.07)
MCHC (g/dl)	47.1 ± 0.5	45.1 ± 0.4^*^	43.9 ± 0.4^**^	45.2 ± 0.5	45.2 ± 0.4	46.2 ± 0.5
	46.80 (2.4)	45.45 (1.98)	44.00 (1.52)	44.25 (1.98)	44.85 (2.05)	46.65 (2.15)
WBCs (103/μl)	11.7 ± 0.5	15.2 ± 1.1^**^	13.9 ± 1.7	11.7 ± 1.9	10.3 ± 1.5^†^	9.8 ± 2.2^†^
	11.24 (3.67)	14.10 (7.07)	13.77 (5.53)	10.69 (4.69)	9.39 (4.65)	7.80 (5.04)

Values are mean ± SEM and median (IQR) for the control, dehydrated, and rehydrated camels. Significant difference from control is denoted by ^*^p < 0.05, ^**^p < 0.01 and ^***^p < 0.001, and from dehydrated (day 20) is denoted by ^†^p < 0.05, ^††^p < 0.01, and ^†*††*^p < 0.001. HCT, hematocrit; Hb, hemoglobin; RBCs, Red blood cells; MCV, mean corpuscular volume; MCH, mean corpuscular hemoglobin; MCHC, mean corpuscular hemoglobin concentration; WBCs, white blood cells; and IQR, interquartile range.

### Biochemical findings

The biochemical parameters are presented in Table 5. TP and albumin were significantly increased (*p* < 0.001) after 20 days of dehydration compared to the control. Rehydration caused a progressive and significant drop in TP and albumin at 12 h (*p* < 0.05) and 24 to 72 h (*p* < 0.01) compared to dehydration level matching that of the control value. ALT activity was not affected by dehydration. By 12 h of rehydration, ALT values increased significantly (*p* < 0.01) compared to the control but gradually returned to control levels. AST displayed no significant change during the dehydration and rehydration periods. LDH activity showed a significant increase (*p* < 0.05) with dehydration compared to controls. During rehydration, LDH activity fluctuated but showed no significant difference from both dehydrated and control values. CK decreased significantly (*p* < 0.001) due to dehydration compared to the control. During rehydration, CK activity showed significant increases at 12, 48, and 72 h compared to dehydration values but fluctuated within the control levels. Dehydration for 20 days caused a significant increase (*p* < 0.001) in GGT activity. Thereafter, the GGT activity returned to control values with a significant difference (*p* < 0.05) from the dehydrated group at 72 h of treatment.

**Table 5:** Changes in TP, albumin, ALT, AST, LDH, CK, and GGT enzymes in serum of control (day 20), dehydrated (day 20), and rehydrated (day 20 and after 12, 24, 48, and 72 h rehydration) camels.

**Parameter**	**Control**	**Dehydrated**	**12h rehydrated**	**24 h rehydrated**	**48 h rehydrated**	**72 h rehydrated**
TP (g/dl)	6.0 ± 0.1	7.6 ± 0.1^***^	6.7 ± 0.3^†^	6.4 ± 0.1^††^	6.1 ± 0.2^††^	6.2 ± 0.2^††^
	6.01 (0.53)	7.63 (0.53)	6.88 (1.54)	6.42 (0.65)	6.05 (0.72)	6.27 (0.91)
Albumin (g/dl)	3.3 ± 0.1	4.0 ± 0.1^***^	3.6 ± 0.2^†^	3.3 ± 0.1^††^	3.1 ± 0.2^††^	3.2 ± 0.2^††^
	3.22 (0.53)	3.94 (0.75)	3.75 (0.90)	3.40 (0.48)	3.33 (0.60)	3.39 (0.62)
ALT (IU/l)	10.9 ± 0.0.4	11.0 ± 0.9	13.8 ± 1.1^**^	11.4 ± 1.1	10.6 ± 1.1	10.9 ± 1.1
	9.9 (3.10)	9.8 (4.15)	13.95 (4.38)	11.30 (4.98)	10.00 (3.95)	10.50 (5.38)
AST (IU/l)	115.8 ± 7.5	112.4 ± 9.7	116.1 ± 18.9	106.1 ± 13.1	95.3 ± 11.4	96.3 ± 8.2
	105.30 (31.60)	110.55 (43.02)	114.29 (70.95)	102.25 (34.63)	89.30 (42.25)	95.75 (30.77)
LDH (IU/l)	525.6 ± 19.2	619.6 ± 51.8^*^	631.7 ± 70.5	591.3 ± 61.3	640.8 ± 75.1	645.0 ± 51.5
	490.00 (78.00)	552.50 (125.25)	601.50 (250.25)	550.00 (294.00)	542 (310.00)	596.00 (244.00)
CK (IU/l)	132.0 ± 5.2	93.4 ± 6.9^***^	130.3 ± 20.6^**†**^	110.8 ± 6.7	129.60 ± 14.6^†^	148.5 ± 17.54^††^
	133.500 (43.25)	87.00 (44.00)	140.50 (52.25)	113.00 (26.75)	150.00 (59.00)	151.00 (67.00)
GGT (IU/l)	8.2 ± 0.4	12.3 ± 1.0^***^	12.0 ± 1.9	10.2 ± 1.9	9.5 ± 2.0	7.5 ± 1.0^**†**^
	8.00 (3.00)	11.00 (4.00)	10.00 (7.00)	9.00 (7.00)	17.67 (8.00	7.50 (4.00)

Values are mean ± SEM and median (IQR) for the control, dehydrated, and rehydrated camels. Significant difference from control is denoted by ^*^p < 0.05, ^**^p < 0.01, and ^***^p < 0.001, and from dehydrated (day 20) is denoted by ^†^p < 0.05 and ^††^p < 0.01. TP, total protein; ALT, alanine aminotransferase; AST, aspartate aminotransferase; LDH, lactic dehydrogenase; CK, creatine kinase; GGT, gamma-glutamyl transferase; and IQR, interquartile range.

### Serum minerals

Serum mineral levels are presented in Table 6. Serum Ca increased significantly (*p* < 0.001) with dehydration compared to the control. Then, these levels decreased significantly with rehydration at 12 (*p* < 0.05), 24 (*p* < 0.01), 48 (*p* < 0.001), and 72 (*p* < 0.01) h, attaining the control value within these periods. Serum phosphorus (P) showed a significant increase (*p* < 0.001) with dehydration and at 12 h of rehydration compared to the control. Between 24-72 h post rehydration, p-levels decreased significantly (*p* < 0.001) compared to the dehydration levels but within the control value. Serum Cu levels showed slight elevation with dehydration but decreased significantly (*p* < 0.05) compared to the dehydration levels at 48 and 72 h post rehydration. Dehydration caused a small, non-significant increase in Fe concentration, but values increased significantly (*p* < 0.01) 12 h post rehydration compared to the control but not to the dehydrated group. There were no significant changes observed between the treatments during the remaining period.

**Table 6:** Changes in Ca, P, Cu, and Fe of control (day 20), dehydrated (day 20), and rehydrated (day 20 and after 12, 24, 48, and 72 h) camels.

			**Rehydrated**			
**Parameter**	**Control**	**Dehydrated**	**12 h**	**24 h**	**48 h**	**72 h**
Ca (mg/dl)	9.0 ± 0.1	9.6 ± 0.1^***^	9.0 ± 0.2^†^	9.2 ± 0.1^††^	8.9 ± 0.1^†*††*^	9.0 ± 0.1^††^
	9.02 (0.60)	9.60 (0.75)	8.85 (0.90)	9.2 (0.23)	9.0 (0.20)	8.95 (0.33)
P (mg/dl)	4.7 ± 0.2	7.5 ± 0.3^***^	7.0 ± 0.4^***^^††^	5.1 ± 0.3^†*††*^	4.3 ± 0.2^†*††*^	4.2 ± 0.2^†*††*^
	4.70 (1.50)	7.45 (0.98)	6.75 (1.92)	4.75 (1.30)	4.10 (0.65)	4.15 (0.70)
Cu (μ/dl)	88.1 ± 3.8	97.0 ± 4.1	87.2 ± 8.6	87.2 ± 6.4	84.2 ± 3.8	81.0 ± 6.7^†^
	90.00 (30.00)	96.00 (16.25)	86.00 (35.25)	87.50 (23.50)	83.00 (16.00)	75.50 (26.25)
Fe (μg/dl)	79.6 ± 5.4	89.4 ± 8.4	99.4 ± 3.4^**^	104.4 ± 9.8	83.5 ± 5.0	94.0 ± 6.9
	72.80 (41.90)	92.50 (42.52)	97.60 (8.67)	98.65 (49.98)	83.40 (18.50)	88.60 (18.90)

Values are mean ± SEM and median (IQR) for the control, dehydrated, and rehydrated camels. Significant difference from control is denoted by ^**^p < 0.01 and ^***^p < 0.001, and from dehydrated (day 20) is denoted by ^†^p < 0.05, ^††^p < 0.01 and ^†*††*^p < 0.001. Ca, calcium; P, phosphorus; Cu, copper; Fe, iron; and IQR, interquartile range.

### Serum hormones

Hormone levels are presented in Table 7. The plasma progesterone level in control camels was (0.03 ± 0.01 ng/ml), and this showed a 9-fold increase (0.28±0.02 ng/ml) after 20 days of dehydration (*p* < 0.001). In the rehydrated camels, progesterone values decreased significantly after 12 h (*p* < 0.001). Plasma testosterone level in the control camels was (0.22 ± 0.04 ng/ml), and after 20 days of dehydration the testosterone concentration was significantly reduced (*p* < 0.001) to 0.03±0.01 ng/ml and remained low at 12 h (*p* < 0.001), and 24 h (*p* < 0.01) post rehydration. However, testosterone levels increased significantly by 48 h and 72 h (*p* < 0.001) of rehydration compared to dehydration and control values. Dehydration for 20 days induced a significant decrease in fT_3_ (*p* < 0.001). Rehydration caused a gradual and significant rise (*p* < 0.001)in fT3 compared to dehydration values to match control levels 24 h onwards. Dehydration for 20 days induced a significant decrease (*p* < 0.001) in plasma fT4. Yet, plasma fT4 was significantly low at 12 (*p* < 0.001), 24 (*p* < 0.001), 48 (*p* < 0.05), and 72 h after rehydration compared to the control. However, it was significantly increased at 72 h compared to the dehydrated camels.

**Table 7:** Changes in progesterone, testosterone, fT_4_, and fT_3_ in plasma of control (day 20), dehydrated (day 20), and rehydrated (day 20 and after 12, 24, 48, and 72 h) camels.

**Parameter**	**Control**	**Dehydrated**	**12 h rehydrated**	**24 h rehydrated**	**48 h rehydrated**	**72 h rehydrated**
prog. (ng/ml)	0.03 ± 0.01	0.28 ± 0.02^***^	0.06 ± 0.02^†*††*^	0.04 ± 0.01^†*††*^	0.03 ± 0.01^†*††*^	0.04 ± 0.01^†*††*^
	0.028 (0.006)	0.30 (0.07)	0.045 (0.01)	0.03 (0.001)	0.03 (0.005)	0.03 (0.006)
testos. (ng/ml)	0.22 ± 0.04	0.03 ± 0.01^***^	0.03 ± 0.01^***^	0.10 ± 0.06^**†*††*^	0.30 ± 0.09^***, †*††*^	0.30 ± 0.06^***^, ^†*††*^
	1.44 (0.27)	0.025 (0.00)	0.025 (0.02)	0.04 (0.010)	0.026 (0.06)	0.28 (0.09)
fT_3_ (pmol/l)	3.6 ± 0.13	1.73 ± 0.15^***^	2.9 ± 0.17^**^ ^†*††*^	3.15 ± 0.17^†*††*^	3.15 ± 0.19^†*††*^	4.30 ± 0.28^†*††*^
	3.67 (1.08)	1.66 (0.63)	2.92 (0.58)	3.15 (0.52)	3.15 (0.58)	4.40 (1.15)
fT_4_ (pmol/l)	19.11 ± 0.59	11.12 ± 0.92^***^	11.31 ± 0.57^***^	12.44 ± 0.64^***^	13.36 ± 1.18^*^	15.81 ± 0.92^*^^††^
	20.47 (5.79)	12.10 (2.79)	11.35 (2.40)	12.55 (2.51)	13.53 (4.85)	15.39 (3.91)

Values are mean ± SEM and median (IQR) for the control, dehydrated, and rehydrated camels. Significant difference from control is denoted by ^*^p < 0.05, ^**^p < 0.01, and ^***^p < 0.001, and from dehydrated (day 20) is denoted by ^††^p < 0.01 and ^†*††*^p < 0.001. prog., progesterone; testos., testosterone; fT3, free triiodothyronine; fT4, free thyroxine; and IQR, interquartile range.

### Serum vitamins

Vitamin levels are presented in Table 8. Vitamin A concentration was significantly increased by dehydration (*p* < 0.001). Rehydration values of Vitamin A were increased at 12 (*p* < 0.001), 24 (*p* < 0.05), and 48 (*p* < 0.05) h compared to control but not to dehydration values. Control values were attained at 72 h post rehydration. Dehydration caused a significant increase (*p* < 0.001) in serum levels of Vitamin B1; however, these values progressively and significantly decreased compared to dehydration (*p* < 0.001) and control levels (*p* < 0.001) throughout the rehydration period. Dehydration for 20 days caused a significant increase (*p* < 0.001) in Vitamin E levels compared to the control. The levels of Vitamin E decreased significantly throughout the rehydration period (*p* < 0.001) compared to dehydration values but attained the control level from 12 h onwards (Table 8).

**Table 8:** Changes in Vitamin A, Vitamin B1, and Vitamin E in the serum of control (day 20), dehydrated (day 20), and rehydrated (day 20 and after 12, 24, 48, and 72 h) camels.

**Parameter**	**Control**	**Dehydration**	**12 h rehydrated**	**24 h rehydrated**	**48 h rehydrated**	**72 h rehydrated**
Vit. A (mg/l)	0.3 ± 0.01	0.4 ± 0.011^***^	0.7 ± 0.1^*^	0.5 ± 0.06^*^	0.5 ± 0.05^*^, ^††^	0.41 ± 0.08
	0.30 (0.08)	0.40 (0.06)	0.67 (0.40)	0.57 (0.23)	0.55 (0.18)	0.33 (0.29)
Vit. B1 (μg/l)	28.1 ± 1.4	45.5 ± 1.5^***^	20.3 ± 1.3^***^^†*††*^	17.9 ± 1.9^***^^†*††*^	15.5 ± 0.6^***^^†*††*^	14.2 ± 1.3^***^^†*††*^
	29.10 (8.62)	45.64 (9.65)	19.64 (5.03)	16.64 (7.19)	15.82 (2.36)	12.73 (1.11)
Vit. E (mg/l)	0.34 ± 0.03	0.84 ± 0.10^***^	0.35 ± 0.03^†*††*^	0.31 ± 0.01^†*††*^	0.30 ± 0.02^†*††*^	0.28 ± 0.02^†*††*^
	0.31 (0.16)	0.78 (0.23)	0.36 (0.09)	0.296 (0.05)	0.30 (0.05)	0.28 (0.04)

Values are mean ± SEM and median (IQR) for the control, dehydrated, and rehydrated camels. Significant difference from control is denoted by ^*^p < 0.05 and ^***^p < 0.001, and from dehydrated (day 20) is denoted by ^**††**^p < 0.01 and ^**†*††***^p < 0.001. Vit. A, Vitamin A; Vit. B1, Vitamin B1; Vit. E, Vitamin E; and IQR, interquartile range.

### Kidney gene expression

Using tissue harvested from the same animals used in this study, we have previously cataloged the transcriptomes of the dromedary camel kidney cortex and medulla and have described how these change with dehydration and subsequent rehydration (7). We have mined these datasets to explore the relative expression of transcripts encoded by genes relevant to the findings of this study (Table 9). We found that the expression of some genes was up-regulated by dehydration, whereas some were down-regulated. Whilst some of these genes returned to control levels following rehydration, others did not and maintained an altered expression level during recovery. The physiological relevance of these observations is addressed in the discussion.

**Table 9:** Expression of genes detected by RNA-seq in kidney cortex (C) and medulla (M) during dehydration (day 20) and rehydration (day 23) relative to control (day 20); significance between groups is *p* < 0.01.

**Gene**	**Protein coded**	**Kidney (C: cortex; M: medulla)**	**Gene expression control**	**Gene expression dehydrated**	**Gene expression rehydrated**
*ACSBG1*	Acetyl coenzyme A synthetase	M	1	106.11	15.22
		C	1	10.24	1.21
*ANGPTL4*	Encodes for protein increase in lipolysis during fasting, glucocorticoid dependent	M	1	6.34	7.00
		C	1	7.04	4.31
*CYP2A13*	Encodes for Cytochrome oxidase enzymes, oxidation/reduction processes (cholesterol, lipid, steroid)	C	1	18.90	4.42
*FADS1*	Encodes for enzyme involved in the saturation of fatty acids	M	1	0.33	0.62
		C	1	0.24	1.02
*FADS2.1*	Encodes for enzymes involved in the saturation of fatty acids	C	1	0.23	0.78
*FADS2*	Encode for enzyme involved in the saturation of fatty acids	M	1	0.2	0.37
*THRSP*	Encodes for thyroid hormones inducible in Hepatic protein	C	1	0.14	0.47
*OMD*	Encodes for protein involved in osteomodulation	M	1	5.47	1.14
*CA9*	Encodes for carbonic anhydrase isozyme involved in acid-base balance/bone resorption etc.	C	1	3.80	2.24
*CAPS*	Ca ion binding protein for transport	C	1	1.05	2.86

## Discussion

During a period of dehydration, the dromedary camel generally needs to adapt to the stress of thirst and to the energy shortage as a consequence of anorexia (33). In this study, we have described the effects of dehydration and rehydration on oxidative stress markers, serum biochemical and hematological parameters, and tissue morphology in the dromedary camel. Dehydrated camels showed loss of body weight. We also observed reduced urine volume and frequency, with a change in urine color and consistency, and passage of very dry feces during the experimental period as a consequence of dehydration, which confirms previous reports (11, 13–15, 34). Reduced metabolic rate had been previously reported during dehydration (12, 13). The dromedary camel normally passes copious saliva to dilute and buffer the very dry food for easy digestion; however, the microscopic appearance of the parotid salivary gland in this study depicted the acini to be devoid of serous secretions, which could be a direct result of dehydration.

The dehydrated camels showed anorexia with a significant decrease in blood glucose concentration (unpublished data) and mostly seemed to rely on body fat mobilization for energy supply. In this respect, the dehydrated camels showed reduced hump size, displayed gelatinization of kidney and cardiac fat, in addition to the change in expression of genes (*ANGPTL4, ACSBG1, FADS 1&2, THRSP*) involved in fat metabolism. Protein catabolism seemed not to be a major source, as CK activity was reduced during dehydration. Generally, the body relies on burning fat stores first prior to protein in case of energy shortage (17). Taking the kidney as an example of an active and vital organ during dehydration with stored fat outside its tissues (Table 9), RNA-Seq analyses of genes showed that there was an increased expression in the genes encoding proteins involved in energy production and decreased expression of genes encoding for energy expenditure. For example, the *ANGPTL4* gene, encoding the glucocorticoid-dependent protein that increases lipolysis during fasting (35), showed a significant increase in expression from basal levels following dehydration, supported by a high concentration of cortisol in the plasma of the dehydrated camels. The *ACSBG1* gene, encoding acetyl coenzyme A synthase that catalyzes the formation of acetate from long-chain fatty acids for energy production, also showed very high expression in the kidney cortex and medulla after dehydration. On the other hand, there was down-regulation of *THRSP* gene expression, encoding for proteins involved in thyroid hormone production and energy metabolism, mirrored by low plasma fT3 and fT4 concentrations. Furthermore, genes *FADS 1&2*, involved in the conversion of unsaturated to saturated fatty acids, exhibited a low expression.

Energy supply from fat mobilization and protein degradation is a vital process for the survival of dromedary camels during dehydration as well as rehydration without causing serious metabolic problems. Normally, the stress of dehydration and anorexia is expected to induce hepatic lipidosis (fatty liver)/ketosis in the dromedary as a result of a high level of fat mobilization similar to cattle, sheep, llamas, and alpacas (19, 20, 36). However, ketosis has not been reported in dromedary camels even after starvation (37, 38), as the camel has a very low β hydroxybutyrate dehydrogenase, a key enzyme in ketone body formation in liver and rumen epithelium compared to true ruminants (39, 40). Also, we have not demonstrated hepatic lipidosis in gross examination microscopy in liver cells nor encountered offensive smells of ketosis in camel breath, sweat, and urine.

Cortisol, a glucocorticoid hormone, is synthesized and released by the adrenal cortex, both of which are increased by stress, whereas catecholamines (epinephrine and norepinephrine) are secreted by the adrenal medulla with different effects (41–43). Epinephrine acts on beta receptors in the heart, lungs, pancreas, and arteries of skeletal muscles to form glucose to boost body energy and to increase heart rate, respiratory rate, sweating, etc. Moreover, it acts on the pancreatic receptors to down-regulate insulin production. However, norepinephrine acts on alpha receptors in arteries to trigger their contraction and redirect blood to the main muscles. Dopamine, a neurotransmitter produced in the brain, induces feelings of happiness and motivation by promoting vital processes. Serotonin, however, is another neurotransmitter produced in many parts of the body, including the gastrointestinal tract, central nervous system, skin, and pulmonary neuroendocrine cells. Normally, it mediates the release of nitric oxide and inhibits norepinephrine so as to dilate blood vessels (41, 42, 44).

Dehydration seemed to modulate stress hormones in dromedary camels to serve water preservation and energy production purposes, where a remarkable increase of cortisol, norepinephrine, and dopamine with a simultaneous decrease in epinephrine and serotonin was reported in plasma. It is well known that the increased level of cortisol during stress increases gluconeogenesis and glycogenesis to elevate blood glucose, which has a high demand during anorexia associated with dehydration (41, 45, 46). The rise of norepinephrine levels during the stress of dehydration was thought to increase arterial contractions and redirect blood to major muscles to supply the body with the required energy to keep these animals active (47). At the same time, serotonin's effects on the dilation of blood vessels were reduced. The drop of epinephrine levels in dehydrated camels seemed to serve to reduce water loss by sweating and energy expenditure during dehydration. Dehydration has been reported to cause increased respiratory rate, reduced expiration water loss, and reduced metabolic rate in dromedary camels (1, 11, 12). Further, it has been shown that dehydration and heat stress compromised cardiac output and stroke volume in human subjects (48). The rise of dopamine levels in dehydrated camels was not surprising. This was perhaps an attempt to regulate metabolic processes during dehydration. Dopamine has been successfully used to treat reduced blood pressure, reduced cardiac output, and reduced blood flow to vital organs, as well as to help regulate fluid and electrolyte balance (44). On the other hand, short-term rehydration served to restore cortisol and serotonin to control values and caused a drop in norepinephrine and dopamine concentrations.

Insulin-like growth factor-1 is mainly synthesized and secreted by the liver into the circulation, bound to IGFBP-3 to mediate the growth hormone action in tissues and promote cell growth and differentiation (49, 50). IGF-I down-regulates insulin secretion, occupies insulin receptors, lowers glucose formation in the liver, and increases the synthesis of skeletal muscle protein (50, 51). Dehydrated camels showed decreased plasma IGF-1 and IGF1BP-3 receptor levels, which may reflect a disturbance in growth hormone production during dehydration (52) and probably increased glucose formation. In addition, it is possible that the reduced food intake and low metabolic rate reported in dehydrated camels (11–15, 34), along with the mild degenerative changes encountered in hepatocytes due to dehydration mitigated the liver's ability to produce IGF-1. Rehydration caused a further reduction in IGF-1 and receptor levels in plasma, which might indicate the priority to restore vital organ function to the catabolic effect in muscles.

Normally, the body balances the rate of superoxide, peroxides, and triple oxide formation and their clearance by antioxidants (7, 86–88). However, excess production of these reactive oxygen species (ROS) during stress conditions leads to OS with the production of hydroxyl radicals (OH) and peroxynitrite oxidant (ONO2), which cause peroxidation of lipids, oxidation of proteins, and damage of cellular DNA (53). Malondialdehyde and 4-hydroxy-2-nonenal are formed from lipid peroxidation alongside nitric oxide (NO) and nitrogenous substances from oxidation and destruction of proteins (54, 55). Similarly, the body has major antioxidants, such as reduced glutathione (GSH), superoxide dismutase (SOD), and catalase (CAT) enzymes, and Vitamins A, B1, C, and E, which scavenge ROS and confer protection to cells (8, 56). GSH is oxidized to GSSS and depleted by reducing free radicles such as hydroxyl ions (OH-). Vitamin A is obtained from feed and stored in the liver to play vital roles, including antioxidant effects (57–59). Vitamin B1 is involved in glucose metabolism, ROS regulation, and acetylcholine generation for nerve transmission (60, 61). The dromedary camel meets its thiamine requirements from microbial synthesis (62), transports it bound to specific transport proteins and RBCs, and excretes it in urine (61). Vitamin E is a potent antioxidant that is essential in reproduction, cellular respiration, prostaglandin synthesis, etc. (8, 63). Roughages are the main sources of vitamins in ruminants. Vitamin E is transported in association with lipoprotein, and its metabolites excreted in bile (63). Significant increases in MDA, NO, GSH, and CAT have been reported in the abomasal mucosa (6) and kidney cortex (25) of dehydrated camels.

In this study, the dehydrated camels showed tissue OS as expressed by high MDA in the liver and plasma and a mild degree of degenerative changes in hepatocytes. At the same time, the dehydrated camels seemed to develop a potent antioxidant system, which increased simultaneously in the liver (GSH) and serum (Vitamins A, B1, and E) with MDA to confer tissue protection and possibly to ease OS effects. On the other hand, rehydration restored GSH and MDA in the liver with a gradual drop in vitamin levels, possibly due to depletion by ROS with little compensation from food. Vitamin B1 attained subclinical values at the end of the rehydration period, similar to that reported in animals with subclinical vitamin deficiency (64). The low levels of Vitamin B1 can seriously jeopardize the health of these animals and may possibly lead to poleoencephalomalacia, a fatal condition in camels, if not promptly treated.

Progesterone is a steroid reproductive hormone synthesized in the ovaries and placenta, but low levels are produced by males (65). The hormone metabolizes fat involved in thermogenesis and may enhance ketone body formation in the liver, besides other functions (66). The extremely high progesterone level in dehydrated camels seemed to be related to energy mobilization from fat. Further, the expected increase in body temperature of camels during dehydration (12, 33) might be partially induced by the high progesterone/norepinephrine through thermogenic function, as seen during ovulation (67). Progesterone increased by rehydration might possibly be another mechanism for water conservation by raising body temperature to minimize evaporative cooling; however, it does not appear to be involved in ketosis. Progesterone quickly returned to control values after rehydration.

Testosterone displays a ten-fold increase during the rutting season in dromedary camels (68). Testosterone and cortisol play a significant role in the metabolism of carbohydrates and proteins (42). We observed a significant drop in plasma testosterone and a significant elevation in plasma cortisol in dehydrated camels; however, the control levels of both hormones were attained with rehydration. The cause of the major drop in testosterone during dehydration and early rehydration might be due to the high cortisol levels, which antagonize and block testosterone receptors (42) during dehydration and/or to the reduced basal metabolic rate (42, 43).

FT3 and fT4 perform a number of metabolic functions, including increased basal metabolic rate, carbohydrate metabolism, fat lipolysis, cholesterol reduction, etc. Dehydration caused a significant decrease in both fT3 and fT4, with fT4 levels being more affected. Decreased total thyroid hormones (TT3, TT4) due to dehydration have been reported to cause reduced metabolic rate and OS and minimize respiratory water loss (69, 70). Recently, we reported reduced cellular membrane cholesterol in kidneys to be an essential factor in cellular water conservation (7). This finding is in line with the reduced biologically active free thyroid hormones that we reported. Factors such as hypovolemia (71), stress (72), and the effects of dehydration on gut bacteria seemed to be implicated in thyroid hormone reduction. Again, factors lowering the conversion of T4 to T_3_ in tissues, such as high levels of cortisol (73), catecholamine (74), and pro-inflammatory cytokines (75), might be involved as well. In this study, we demonstrated low expression of the thyroid hormone-responsive gene *THRSP* in kidney tissue due to dehydration. FT3 and fT4 levels were restored by rehydration with a noticeable improvement in the gene expression.

Ca is involved in bone mineralization and acts as a messenger of signal transduction, producing cell stress and damage. Besides bone mineralization, P is also involved in the phosphorylation process of molecules in the pro-inflammatory and anti-inflammatory transcription factor pathways (76). The link between increased serum Ca and P and dehydration is not clear; however, this may be related to the kidney. During the stress of dehydration, the kidney is actively involved in water regulation via renin-angiotensin11-aldosterone as well as in the process of Ca/P metabolism via 1,25 (OH)2-vitamin D3 formation and acid-base balance regulation (77). Although we have not measured the hormones involved, we were able to report activation of gene expression during dehydration (Table 9). Such increased gene expression included the *OMD* gene (encoding a protein involved in osteomodulation), the *CA9* gene (encoding carbonic anhydrase involved in acid-base balance and bone resorption), the *PCDH8* gene (encoding a protein involved in Ca ion binding), and the *CAPS* gene (encoding a Ca transporter protein). Cu and Fe are prosthetic groups in the antioxidant enzymes CAT, SOD, as well as cytochrome oxidase, which are involved in OS (38, 78). Serum Cu and Fe levels were increased, perhaps due to the increased plasma protein transporters and/or possibly engaged in the antioxidant enzyme synthesis to help combat OS.

There are many discrepancies regarding the effects of dehydration on AST, ALT, CK, LDH, and GGT serum enzymes (23, 79, 80). The enzymes AST and ALT are important in the synthesis and degeneration of amino acids and in energy production in the Krebs cycle (81, 82). AST and ALT leak in serum in case of acute hepato-renal and/or cardio-skeletal cell damage (38). Since there were no acute or striking histopathological changes induced by dehydration or rehydration in liver and kidney cells and cardiomyocytes of the dehydrated and rehydrated camels, it is logical to see only a small or no elevation in the activities of these two enzymes. CK and LDH enzymes rise in cardiac and skeletal muscle injury (38). CK activity dropped significantly by dehydration, reflecting decreased enzyme synthesis. However, CK decrease was only temporary, and animals attained control enzyme activity 12 h after rehydration, indicating that OS was unlikely to have occurred in heart and skeletal muscle.

Unlike CK, the LDH activity increased with dehydration, but the source of the enzyme elevation was not clear. It is possible that LDH elevated to catalyze the process of anaerobic oxidation induced by increased respiration rate for a long period to compensate for the reduced O2 tension during exercise and dehydration (83, 84). However, the LDH rise was corrected by rehydration. The main source of GGT in serum is the epithelial cells lining the biliary tract and the smooth endoplasmic reticulum of hepatocytes. Thus, it is a sensitive indicator of hepatobiliary disorders in many species, including camels (85, 86). The mild elevated GGT activity in dehydrated camels might be due to increased hepatobiliary synthesis of the enzyme rather than leakage from the degenerating cells (87). Rehydration quickly decreased and normalized GGT activity. Long-term dehydration is expected to induce physiological cardiomyocyte hypertrophy as a response to an increased load of pumping concentrated blood (88, 89). The physiological myocardial hypertrophy is controlled by molecules and key enzymes in the PI3K/AKT/mTOR/FOXOs signaling pathways (88, 90). It is worth noting that we observed only sporadic cardiomyocytes that displayed hypertrophy at the end of the dehydration period, but these degenerative changes were eventually reversed by rehydration. Also, there were no major effects in the key enzymatic signaling pathways for physiological hypertrophy (unpublished data) along with normal ALT and AST and decreased CK activities, indicating no major effects induced to cardiomyocytes by dehydration.

The increases in HCT, Hb, MCV, MCH, and WBC, and the decrease in MCHC values reported during long-term dehydration were somewhat different from previous studies (91–93), where non-significant increases were reported. The possible explanation for this could be that our study was performed for a longer period and at high ambient temperature, whereas the previous studies were conducted for a shorter period and in a cooler environment. Rehydration quickly facilitated the restoration of most of these changes to control levels within 24–72 h. There was a mild and transient lymphocytic increase in the white pulp of the spleen of dehydrated camels, causing lymphocytic hyperplasia, which was observed concurrently with a significant increase in WBC, perhaps to increase the immunity of dehydrated camels. The increased red pulp area of the spleen by rehydration might possibly be a physiological phenomenon resulting from increased blood volume and RBC diameter. It is well known that the dromedary camel RBCs can swell up to 240% of their initial volume without rupture (5). This might cause slow passage of cells and fluids through the narrow endothelial gaps of the venous sinuses of the spleen. Thus, the red pulp of the spleen might possibly be acting as an RBC reservoir during early rehydration. The blood and RBC volume increase is also controlled by the gut, where it retains water for up to 24 h after rehydration to maintain slow fluid equilibrium in the circulatory system, and RBC burst (9, 94).

We concluded that the dromedary camel can tolerate dehydration and associated anorexia better than all other domesticated animals. However, we found that dehydration caused modulation of stress hormones and a versatile system for energy mobilization and metabolism. It also increased oxidative markers mirrored by activation of the antioxidant system. The dehydrated/rehydrated camels induced reduced hump size, anorexia, and gelatinization of fats in vital organs, indicating fat mobilization to compensate for energy requirement. Dehydration caused minor or no histopathological changes. The changes observed during dehydration serve to minimize water loss and compensate for energy shortage induced by the accompanying anorexia. The body's vital functions were quickly refurbished by rehydration.

## Limitations of this study

We did our best to control the experimental work; however, some variations were inevitable. The body weights were not directly measured as no large animal balance was available, which forced us to calculate these weights by measurements using standard formulas with potential inborn errors. We did our very best to collect and preserve specimens on ice, dry ice, or liquid nitrogen as quickly as possible after slaughtering the camels in the abattoir. Keeping specimens at −20°C or at −80°C before analysis might have caused potential degradation effects on their quality. We are confident that these potential limitations are more or less of a biological nature rather than technical bias to compromise this work.

## Data availability statement

Publicly available datasets were analyzed in this study. This data can be found here: https://www.ncbi.nlm.nih.gov/geo/query/acc.cgi?acc=GSE173683.

## Ethics statement

The study protocol was approved by the Animal Ethics Committee of the United Arab Emirates University with approval ID No: ERA- 2016-4327. During the experimental period all applicable international, national, and/or institutional guidelines for the care and use of animals were followed. We would like to confirm that, this work adhered to the ARRIVE list of guidelines for reporting animal research. The study was conducted in accordance with the local legislation and institutional requirements.

## Author contributions

AAde and DM planned and advised on the conduct of the study, contributed and assembled all data, conceived of the study, and provided general oversight of its conduct. MAl, HA, OA, and KA oversaw the day-to-day progress of the study (caring for and feeding the animals, collecting and delivering blood samples, measuring body weights, and collecting data for analysis) and also contributed to writing the paper. NA, MAd, AS, SA, AAda, and TF performed laboratory analysis of HPLC, Radioimmunoassay technique, and Colorimetric assay and oversaw quality control of the different assays. JK, AE, ZA, and MJ conceived the study, oversaw its completion, provided statistical analysis, and contributed to the writing of the manuscript. All authors contributed to the article and approved the submitted version.
